# Liquid Seal for Temperature Sensing with Fiber-Optic Refractometers

**DOI:** 10.3390/s140814873

**Published:** 2014-08-13

**Authors:** Ben Xu, Jianqing Li, Yi Li, Jianglei Xie, Xinyong Dong

**Affiliations:** 1 Faculty of Information Technology, Macau University of Science and Technology, Macao, China; E-Mail: xubenfiles@163.com; 2 College of Optical and Electronic Technology, China Jiliang University, Hangzhou 310018, China; E-Mails: yli@cjlu.edu.cn (Y.L.); 1208030014@cjlu.edu.cn (J.X.); xydong@cjlu.edu.cn (X.D.)

**Keywords:** fiber-optic refractometer, temperature sensor, liquid seal, thermo-optic effect

## Abstract

Liquid sealing is an effective method to convert a fiber-optic refractometer into a simple and highly sensitive temperature sensor. A refractometer based on the thin-core fiber modal interferometer is sealed in a capillary tube filled with Cargille oil. Due to the thermo-optic effect of the sealing liquid, the high refractive-index sensitivity refractometer is subsequently sensitive to the ambient temperature. It is found that the liquid-sealed sensor produces a highest sensitivity of −2.30 nm/°C, which is over 250 times higher than its intrinsic sensitivity before sealing and significantly higher than that of a grating-based fiber sensors. The sensing mechanisms, including the incidental temperature-induced strain effect, are analyzed in detail both theoretically and experimentally. The liquid sealing technique is easy and low cost, and makes the sensor robust and insensitive to the surrounding refractive index. It can be applied to other fiber-optic refractometers for temperature sensing.

## 1. Introduction

Fiber-optic temperature sensors have received significant attention for their unique advantages such as immunity to electromagnetic interference and fast response, as well as remote sensing capabilities, thus a wide variety of temperature sensing schemes have been proposed and reported to date, such as fiber knot [1,2], high-birefringence fibers (HBFs) [3], fiber Bragg gratings [4,5], long period fiber gratings [6], fiber in-line interferometers [7–9], and so on. Nam *et al.* studied a thin cladding-etched SMF bent to form a small fiber knot; the sensor has a sensitivity of 0.212 nm/°C by utilizing whispering gallery mode resonance [1]. Using HBFs, Liu *et al.* demonstrated a fiber-optic loop mirror (FLM) sensor with a high sensitivity of 0.94 nm/°C [3], but the HBF used was quite long (~72 cm) due to the limited birefringence of conventional fiber. In the case of fiber-grating-based sensors, due to their small thermo-optic and thermal expansion coefficients, their sensitivities are usually low (~0.01 nm/°C for FBG and ~0.1 nm/°C for LPFG). To improve the sensitivity, some techniques such as cladding etching [10], polymer packaging [11], and others [12] have been adopted. However, problems including fragility, complications and high cost still exist. The fiber in-line interferometer sensors, such as multimode- [7], Fabry-Perot- [8], Michelson- [9] and Mach-Zehnder [13,14], are easy-to-fabricate and low-cost. But their temperature sensitivities are still low (~10–100 pm/°C) due to the small optical path difference (OPD) between the interference modes varying with temperatures. To overcome the above limitations, Qiu *et al.* infiltrated liquids into the micro-holes of the photonic crystal fiber (PCF) of an in-line Mach-Zehnder interferometer (MZI) [15] The injected liquid enlarges the OPD between core modes and high order modes, owing to the large thermo-optic coefficient difference between liquids and silica. Thus, very high temperature sensitivities on the order of several nm/°C have been obtained. Furthermore, higher temperature sensitivities of several decade nm/°C were achieved by using selectively liquid-filled PCFs [16–19]. However, to inject liquids into the micro-holes of these PCF-based sensors is complicated and the RI cross-sensitivity and bending sensitivity cannot be avoided [15].

It is worth noting that some fiber-optic refractometers can be converted into other types of sensors by coating with functional materials whose RI changes with the surroundings. For an example, a refractometer based on a thin-core fiber modal interferometer (TCFMI) was coated with poly (allylamine hydrochloride and acrylic acid) for sensing pH [20]. It can also be coated with PEO for humidity measurements [21]. Meanwhile, the intrinsic temperature sensitivity of bare TCFMI was found to be quite low (less than 20 pm/°C) [13,20,22]. Supposing the coated material has a high thermo-optic coefficient, the refractometer should be sensitive to ambient temperature. An SMS fiber refractometer coated with polymer for temperature sensing with a sensitivity of 15 nm/°C has been proved [23]. However, the sensor was fragile and complicated to fabricate. The multimode fiber must be first etched to a thin residue diameter (~50 μm) and then coated with polymer film.

In this study, liquid sealing is demonstrated as another effective method to convert a fiber-optic refractometer into a highly sensitive temperature sensor. The refractometer based on a TCFMI was sealed in a capillary tube filled with Cargille oil, which has a high thermo-optic coefficient. The experimental results show that the sensitivity of liquid-sealed TCFMI is significantly higher than that measured before sealing. As for the temperature sensing mechanisms, besides of the thermo-optic effect of the sealing liquid, the strain effect resulting from the thermal expansion of the capillary tube and the hydraulic pressure in tube from the liquid volume expansion is also discussed in detail. The capillary tube, acting as the package, makes the sensor robust and protects it from sensitivity to the surrounding RI. The liquid sealing technique is also easy to apply to other fiber-optic refractometers for temperature sensing.

## 2. Sensing Principle

**Figure 1.**
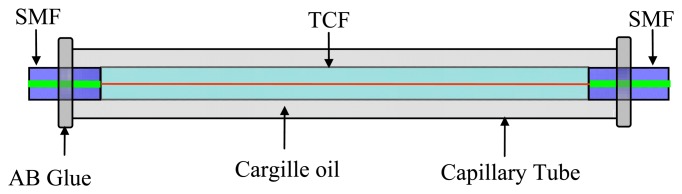
Schematic of the proposed sensor.

Figure 1 shows the schematic of the liquid-sealed fiber-optic refractometer, where the demonstrated refractometer is based on a TCFMI. The TCFMI was firstly constructed by splicing a length of ~20 mm thin-core fiber (TCF, 405HP, Nufern, East Granby, CT, USA) to two telecom single-mode fibers (SMFs, SMF-28, Corning Incorporated, Corning, NY, USA). The used TCF has a smaller core diameter (of ~2.1 μm) than SMF-28 (of ~8.3 μm) while having the same cladding diameter (of ~125 μm). Then, the interferometer was inserted into a silicate capillary tube to ensure the TCF section was totally sealed in the tube, then Cargille oil (Series AA, # 1806X, bought from Cargille Labs, Cedar Grove, NJ, USA) was then injected into the capillary tube. The oil has a nominal RI value of 1.44 and a thermo-optic coefficient of −3.95 × 10^−4^/°C. The capillary tube has a length of ~40 mm, and its inner and outer diameters are ~300 μm and ~600 μm, respectively. By dropping a little oil droplet above the upper port of the capillary tube placed vertically without using any microscope, the oil would infiltrate rapidly into the capillary tube under its own weight and capillarity, removing the air within it. Finally, AB glue was adopted to prevent the sealing-liquid leaking out. The whole liquid sealing process can be done manually and easily.

For the TCFMI, as analyzed in [13,20–22], many cladding modes are excited at the first splice point and propagated in TCF cladding due to the mismatch of core diameters of fibers. Then, they interfere with the core mode of TCF at the second splice point. The TCFMI is a typical fiber in-line MZI, and usually used as an excellent refractometer with low temperature sensitivity [20,22]. Furthermore, its intrinsic temperature sensitivity *S_in_*(*T*) can be expressed as:

(1) $$S_{in}(T)=\frac{d\lambda_{D}}{dT}≅[\frac{\lambda_{D}}{\Delta n_{\text{eff}}}(\frac{\partial \Delta n_{\text{eff}}}{\partial n_{co}}\frac{dn_{co}}{dT}+\frac{\partial \Delta n_{\text{eff}}}{\partial n_{cl}}\frac{dn_{cl}}{dT})+\frac{\lambda_{D}}{L}\frac{dL}{dT}]/(1-\frac{\lambda_{D}}{\Delta n_{\text{eff}}}\frac{\partial \Delta_{\text{eff}}}{\partial \lambda })$$

where λ*_D_* denotes the tracing resonance dip wavelength of the transmission spectrum, *n_co_* and *n_ci_* denote the effective indices of the core and cladding modes of TCF, and Δ*n_eff_* is the difference between them. *dn_co_/dT* and *dn_cl_*/*dT* are the thermo-optic coefficients of core and cladding of TCF, respectively, L and *dL*/*dT* are the length and thermal expansion coefficient of the TCF. Due to the small thermo-optic coefficients (*dn_co_*/d*T* and *dn_cl_*/d*T*, ~10^−5^–10^−6^/°C) and thermal expansion coefficient of TCF (~5 × 10^−7^/°C), the intrinsic temperature sensitivity *S_in_*(*T*) of TCFMI would be inevitably low according to Equation (1), which explains the obtained low sensitivities of less than 20 pm/°C reported in [13,20,22].

Due to the TCFMI being immersed in a liquid, at least two factors influence its transmission spectra when the temperature changes. One is the intrinsic temperature response of the TCFMI, *S_in_*(*T*), as discussed above; the other is the RI response of the TCFMI, *S_SRI_* (*T*), due to its high RI sensitivity and the thermo-optic effect of sealing-liquid. Therefore, the temperature sensitivity *S_se_*(*T*) of liquid-sealed TCFMI can be described as:

(2) $$S_{se}(T)=S_{in}(T)+S_{\text{SRI}}(T)=S_{in}(T)+\frac{d\lambda_{D}}{dn_{\text{liquid}}}\cdot \frac{dn_{\text{liquid}}}{dT}=S_{in}(T)+S(\text{SRI})\cdot \sigma$$

where dλ*_D_*/d*n_liquid_* denotes the RI sensitivity of the refractometer *S*(*SRI*), and *dn_liquid_*/*dT* denotes the thermo-optic coefficient of the sealing-liquid σ. According to Equation (2), the RI sensitivity of the TCFMI can be utilized for its temperature sensing. Furthermore, higher RI sensitivity *S*(*SRI*) and thermo-optic coefficient σ will be benefit for improving the temperature sensitivity of the liquid-sealed TCFMI.

## 3. Experimental Results and Discussion

**Figure 2.**
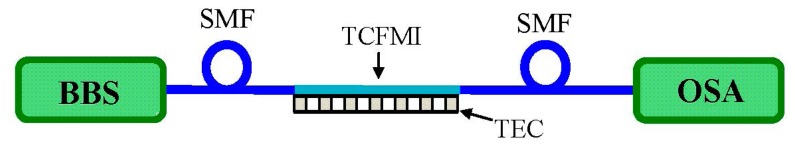
The setup for temperature sensing.

For comparison, the temperature responses of the TCFMI before and after sealing were tested. Figure 2 gives the schematic of the experimental setup for temperature sensing. The sensing device (*i.e.*, the bare or sealed TCFMI) was embedded into a slot of a copper block which was pasted on a thermoelectric cooler (TEC). The temperature of the sensing device can be controlled with a resolution of 0.1 °C. One end of the sensing device is connected to a broadband light source (BBS) with the wavelength range of 1400–1700 nm and the other end to an optical spectrum analyzer (OSA, AQ6370C, Yokogawa, Musashino, Tokyo, Japan) with a resolution of 0.02 nm. It is noted that all our experiments are based on the same one TCFMI with a segment of TCF at ~20 mm.

**Figure 3.**
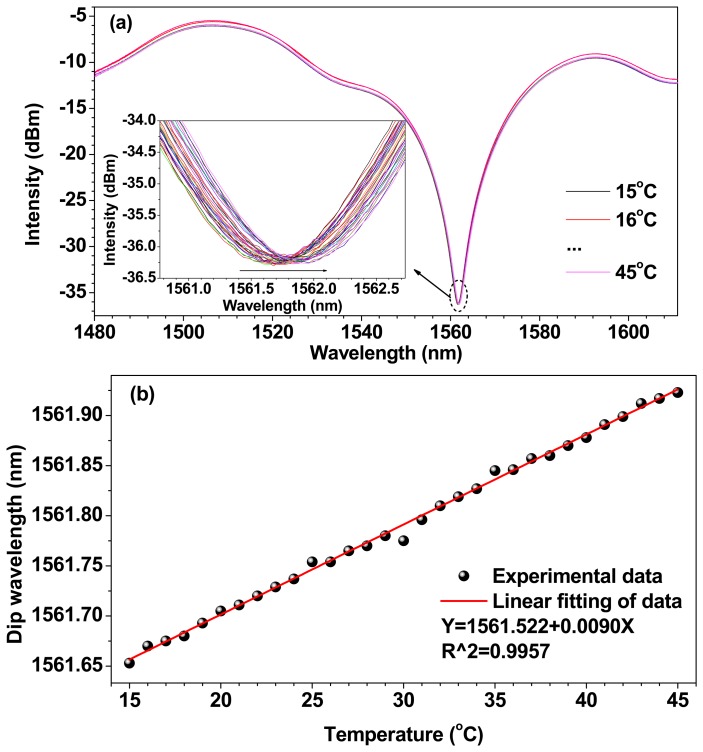
Temperature response of the bare TCFMI in air. (**a**) Transmission spectra at different temperatures. The inset shows the tracing resonance dip; (**b**) Resonance dip wavelength *versus* temperature.

Figure 3 shows the temperature response of the bare TCFMI without being sealed. There is a strong resonance dip in the transmission spectrum with the extinction-ratio of ~30 dB at ~1561.7 nm as shown in Figure 3a. It is found that the resonance dip shifts slightly to longer wavelength with the increase of temperature. Figure 3b gives the measured resonance dip wavelengths at different temperatures and the linear fitting results. The slope of the fitting line, corresponding to the temperature sensitivity of the bare TCFMI, is ~9.0 pm/°C. The measured sensitivity is close to those obtained in [13,20,22] where the TCF used has a larger core diameter of 4.2 μm.

**Figure 4.**
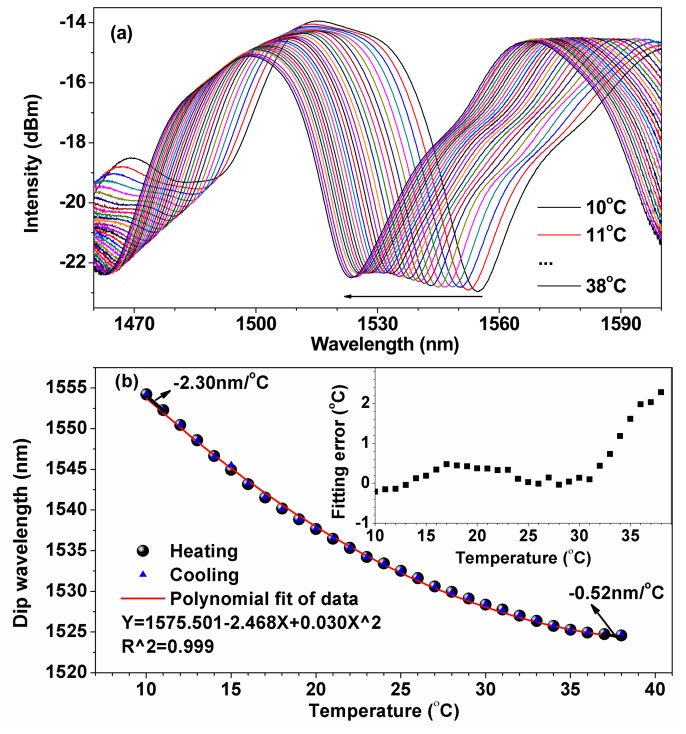
Temperature response of the liquid-sealed TCFMI. (**a**) Transmission spectra at different temperatures; (**b**) Resonance dip wavelength *versus* temperature. The inset gives the fitting error on temperature.

Figure 4 shows the temperature response of the liquid-sealed TCFMI. In our experiments, the sealing-liquid used has a nominal RI value of 1.44, which is corrected to 1.4298 at the operation wavelength of 1550 nm and room temperature (20 °C), and a thermo-optic coefficient of −3.95 × 10^−4^/°C. The capillary tube is made of silicate glass with a length of ~40 mm and sealed with AB glue. The liquid-sealed TCFMI was first heated to 38 °C and maintained there for about 2 h. Figure 4a presents the transmission spectra of the liquid-sealed TCFMI in the heating processing. Tracing the resonance dip with a large extinction-ratio at near 1550 nm, we found that the resonance dip shifts greatly to shorter wavelength with the increase of temperature, contrary to the response of the bare TCFMI. Then, the device was cooled to 10 °C. Figure 4b displays the measured dip wavelengths in both the heating and cooling processes. There was only a slight deviation (<0.1 nm) of the dip wavelength in the cooling process when compared to that in the heating process. The thermal test was repeated several times and the results were quite reproducible.

A second-order polynomial curve was used to fit the dip wavelengths recorded in the heating process across the entire calibration range, and the Adj. R-Square is 0.999. It can be seen that the sensitivity of our sensor increases with the decreasing of temperature. The inset in Figure 4b shows the fitting errors of temperature. It can be seen that, within the tested temperature range, the fitting error increases with the increase of temperature, and its value is less than 1.0 °C within the temperature range of 10–30 °C. And the fitting error can be anticipated to be smaller by using a higher-order polynomial in practice. The maximum sensitivity of the liquid-sealed TCFMI reaches up to −2.30 nm/°C at 10 °C, which is over 250 times higher than that of the bare TCFMI sealed before. Note that the lowest sensitivity of −0.52 nm/°C at 38 °C is also much higher than that of the bare TCFMI. The temperature sensitivity of our sensor is tens or hundreds of times higher than those of fiber grating-based sensors [4–6,10–12] and most interferometric fiber-optic sensors [7–9,13,14,22], and even higher than those of some liquid-sealed PCF-based sensors [15]. In our experiments, the resolution of used OSA is 0.020 nm, then the theoretical temperature resolutions of ~0.008 °C and ~0.038 °C can be achieved at near 10 °C and 38 °C, respectively, for the proposed liquid-sealed TCFMI.

**Figure 5.**
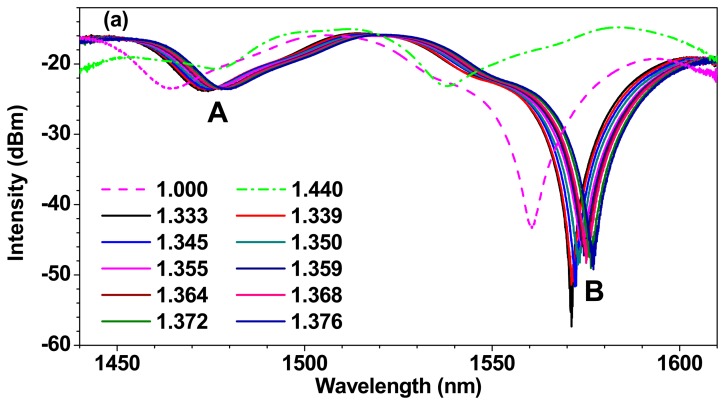

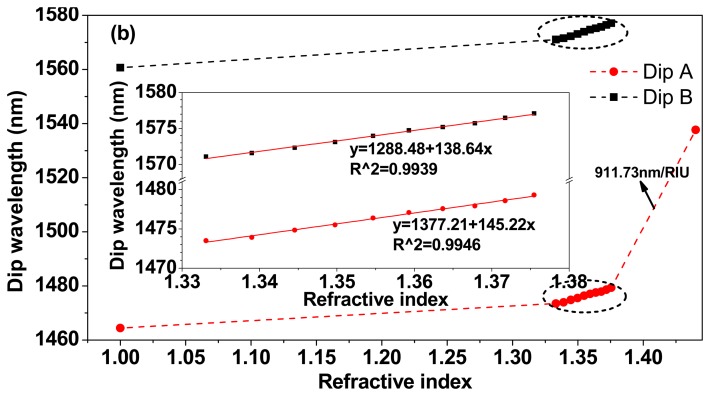
SRI response of the TCFMI. (**a**) Transmission spectra of the TCFMI with different RI solutions; (**b**) Resonance dip wavelength *versus* RI. The inset shows the details of the elliptical area.

To better understand the sensing principle of the liquid-sealed TCFMI, the spectrum blue shift and nonlinear temperature sensitivity will be discussed below. After the liquid-sealed TCFMI was immersed in acetone solution to dissolve AB glue at its two ends, the RI response of the bare TCFMI was tested. Figure 5a gives its transmission spectra in air, salt solutions and Cargille oil at room temperature corresponding to surrounding RIs of 1.000, 1.333–1.376 and 1.440, respectively. There are two resonance dips, A and B, with large extinction-ratios. With the increase of surrounding RI (SRI), they shift to longer wavelength, and finally the dip B shifts out of the focus range. Figure 5b gives the resonance dip wavelengths *versus* SRI. Obviously, for the both dips, their RI sensitivities increase nonlinearly with the increasing of SRI on the whole while they are almost constants within a narrow RI range as shown in the inset (where the red solid lines denote the linear fitting results). Furthermore, for the dip A, its average RI sensitivity is as high as ~911.73 nm/RIU within the SRI range of 1.376–1.440, and its RI sensitivity would be higher for higher SRI according to the change trend shown in Figure 4b. The response to SRI of TCFMI is similar to those LPG-based MZIs [24–26], and the variation of measured SRI sensitivity has been explained in [20]:

(3) $$S(\text{SRI})=\frac{d\lambda_{D}}{dn_{\text{SRI}}}=\frac{-\lambda_{D}}{\Delta n_{\text{eff}}}\frac{\partial n_{\text{eff}}^{cl,j}}{\partial n_{\text{SRI}}}/[1-\frac{\lambda_{D}}{\Delta n_{\text{eff}}}(\frac{\partial n_{\text{eff}}^{co}}{\partial \lambda }-\frac{\partial_{\text{eff}}^{cl,j}}{\partial \lambda })]$$

where *n_SRI_* is the SRI. With the increase of *n_SRI_* but no more than that of the fiber cladding, Δ*n_eff_* will decrease, and then the sensitivity of SRI will increase accordingly. Due to the negative thermo-optic coefficient of sealing-liquid (~ −3.95 × 10^−4^/°C), the temperature sensitivity of the liquid-sealed TCFMI, *S_se_*(*T*), is also negative because the product of *S*(*SRI*) and σ is much larger than *S_in_*(*T*) numerically according to Equation (2), which accounts for the spectrum blue shift and the high temperature sensitivity of the sensor. At the same time, the RI of sealing-liquid increases with the decreasing of temperature. And it would result in nonlinear temperature response because of the nonlinear RI sensitivity of the refractometer.

**Figure 6.**
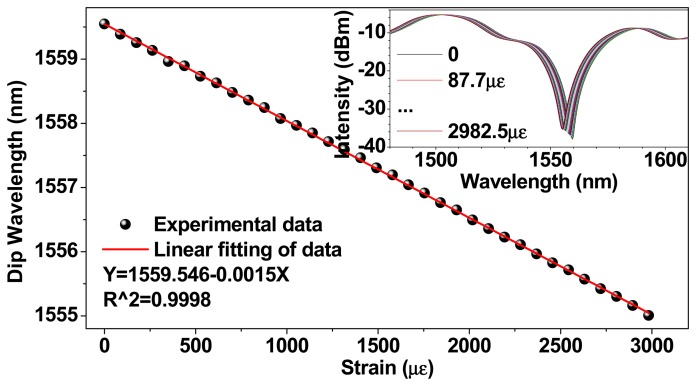
Strain response of the bare TCFMI in air and the inset shows its transmission spectra.

**Figure 7.**
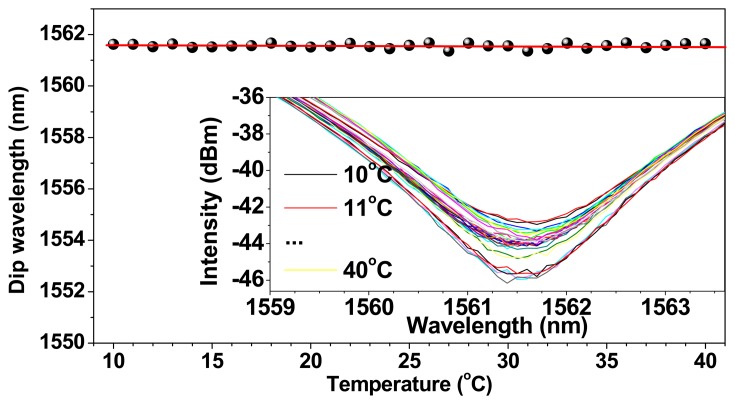
Temperature response of the air-sealed TCFMI. The inset shows the tracing resonance dip.

Additionally, the difference of thermal expansion coefficients between the capillary tube and the used TCF or SMF and the hydraulic pressure in capillary tube from the liquid volume expansion are worth noted, because they would cause longitudinal and axial strain in the TCFMI. For the exemplified liquid-sealed TCFMI, the thermal expansion coefficients of the silicate capillary tube and the fibers used (TCF or SMF) are ~8 × 10^−6^/°C and ~5 × 10^−7^/°C, respectively. To evaluate the strain effect on the sensor, two more tests were carried out as the following. Firstly, the strain response of the bare TCFMI was measured by elongating the interferometer using two linear stages. Then, the bare TCFMI was sealed again in a capillary tube, but was filled with air not liquid, and the temperature response of the air-sealed TCFMI was measured again. The experimental results are shown in Figures 6 and 7, respectively. For the bare TCFMI, the tracing resonance dip presents a blue shift for the increasing strain with a sensitivity of about −1.50 pm/με. For the air-sealed TCFMI, the wavelength of tracing resonance dip fluctuates within ±73 pm around a certain wavelength. The wavelength resolution of the used OSA is 20 pm, which implies that the air-sealed TCFMI is almost insensitive to temperature.

Therefore, the temperature sensitivity of the liquid-sealed TCFMI expressed as Equation (2) can be amended as:

(4) $$S_{se}(T)=S_{in}(T)+S_{\text{SRI}}(T)+S_{S}(T)=S_{in}(T)+S(\text{SRI})\cdot \sigma +S_{s}(T)$$

where *S_s_*(*T*) denotes the temperature sensitivity contribution from the strain. Considering only the thermal expansion coefficient difference between fiber and silicate capillary tube Δα, the longitudinal temperature-induced strain effect can be valuated as Δα/*S_s_*(*S*), where *S_s_*(*S*) is the strain sensitivity of bare TCFMI, with a calculated sensitivity of about −5.0 pm/°C. Actually, additional longitudinal and axial strains would be both induced from hydraulic or baric pressure in capillary tube from the liquid or air volume expansion, and they have both negative sensitivities [27]. Then, the temperature sensitivity contribution from the strains, *S_s_*(*T*) is lightly larger than this calculated value. Figure 7 reveals that *S_s_*(*T*) for air-sealed TCFMI is comparable to the intrinsic temperature sensitivity *S_in_*(*T*) but having contrary spectrum shift. For the liquid-sealed TCFMI, its actual temperature-induced strain effect should be slighter than that for air-sealed one due to its lower thermal volume expansion coefficient of liquid than that of air.

Substituting the measured *S_in_*(*T*) and *S*(*SRI*) into Equation (4), it can be seen that *S_SRI_*(*T*) dominates the final temperature sensitivity of our sensor. By enhancing *S*(*SRI*) and adopting a sealing-liquid of higher σ, *S_se_*(*T*) can be anticipated to be improved. Furthermore, to enhance *S*(*SRI*), some techniques are available, such as selecting sealing-liquids with higher RI or tapering the TCF [28,29].

Furthermore, the limit to *S*(*SRI*) can be estimated according to Equation (4). Supposing *S_in_*(*T*), *S_s_*(*T*) and σ are the order of ~10 pm/°C, ~−10 pm/°C and ~−10^−4^/°C, respectively, *S*(*SRI*) should be higher than 100 nm/RIU to ensure *S_se_*(*T*) is higher than *S_in_*(*T*). It means that, the precondition of liquid seal is that the RI sensitivity of referometer, *S*(*SRI*), is high enough.

Additionally, the response speed of the device is important, which depends on the dimensions and thermal features of the sealing liquid and capillary tube. For the exemplified liquid-sealed TCFMI, the sealing liquid and capillary tube have small volumes of ~2.1 μL and ~6.4 μL, respectively, which ensures a fast response to ambient temperature. Alternatively, a metal capillary tube can be adopted owing to its good thermal conductivity.

## 4. Conclusions

In conclusion, liquid sealing has been demonstrated to be an effective method to convert a refractometer into a simple and highly sensitive low-temperature sensor. Experimental results have shown that the temperature sensitivity of the proposed sensor was significantly enhanced compared to it before sealing and it is much higher than those of fiber grating-based sensors and the most popular interferometric fiber-optic sensors. It was also found that the RI variation of the sealing-liquid dominates the final temperature sensitivity of the sensor due to its high thermo-optic coefficient. The demonstrated liquid sealing technique can be also easily applied to other fiber-optic refractometers for temperature sensing, being especially suitable for refractometers with high SRI sensitivity. The weakness of the liquid sealing technique is that the measurable temperature range is limited to the range between the freezing and boiling temperatures of the liquid used and the outside package.
